# Patient-Generated Collections for Organizing Electronic Health Record Data to Elevate Personal Meaning, Improve Actionability, and Support Patient–Health Care Provider Communication: Think-Aloud Evaluation Study

**DOI:** 10.2196/50331

**Published:** 2025-02-03

**Authors:** Drashko Nakikj, David Kreda, Karan Luthria, Nils Gehlenborg

**Affiliations:** 1 Department of Biomedical Informatics Harvard Medical School Boston, MA United States

**Keywords:** mobile health, patients, electronic health records, sensemaking, data organization, collections, awareness, proactivity, self-advocacy, patient–health care provider communication

## Abstract

**Background:**

Through third party applications, patients in the United States have access to their electronic health record (EHR) data from multiple health care providers. However, these applications offer only a predefined organization of these records by type, time stamp, or provider, leaving out meaningful connections between them. This prevents patients from efficiently reviewing, exploring, and making sense of their EHR data based on current or ongoing health issues. The lack of personalized organization and important connections can limit patients’ ability to use their data and make informed health decisions.

**Objective:**

To address these challenges, we created Discovery, an experimental app that enables patients to organize their medical records into collections, analogous to placing pictures in photo albums. These collections are based on the evolving understanding of the patients’ past and ongoing health issues. The app also allows patients to add text notes to collections and their constituent records. By observing how patients used features to select records and assemble them into collections, our goal was to learn about their preferred mechanisms to complete these tasks and the challenges they would face in the wild. We also intended to become more informed about the various ways in which patients could and would like to use collections.

**Methods:**

We conducted a think-aloud evaluation study with 14 participants on synthetic data. In session 1, we obtained feedback on the mechanics for creating and assembling collections and adding notes. In session 2, we focused on reviewing collections, finding data patterns within them, and retaining insights, as well as exploring use cases. We conducted reflexive thematic analysis on the transcribed feedback.

**Results:**

Collections were useful for personal use (quick access to information, reflection on medical history, tracking health, journaling, and learning from past experiences) and clinical visits (preparation and raising physicians’ awareness). Assembling EHR data into reliable collections could be difficult for typical patients due to considerable manual work and lack of medical knowledge. However, automated collection building could alleviate this issue. Furthermore, having EHR data organized in collections may have limited use. However, augmenting them with patient-generated data, which are entered with flexible richness and structure, could add context, elevate meaning, and improve actionability. Finally, collections might produce a misconstrued health picture, but bringing the physician in the loop for verification could increase their clinical validity.

**Conclusions:**

Collections can be a powerful tool for advancing patients’ proactivity, awareness, and self-advocacy, potentially facilitating patient-centered care. However, patients need better support for incorporating their own everyday data and adding meaningful annotations for future reference. Improvements in the comprehensiveness, efficiency, and reliability of the collection assembly process through automation are also necessary.

## Introduction

### Background

Digital technologies play a pivotal role in facilitating patient-centered care that focuses on understanding patients’ needs and fostering shared decision-making with their health care providers [1]. However, this approach relies heavily on well-informed patients and on effective patient-provider communication [2]. In the United States, a major step toward meeting these requirements was enabling patients to access their medical records from multiple providers through third-party applications [3,4]. While this is a significant achievement, enabling patients to engage in making sense of these data and turn the insights they gain from this process into actionable steps remains a challenge. Consequently, many patients lack a satisfactory understanding of their health, feel discouraged to self-advocate, and have mediocre communication with their providers, which is at odds with the core values of patient-centered care. Therefore, addressing the challenges around sensemaking and the usability of health data will be important to advancing patient-centered care and empowering patients to take an active role in their health journey.

Making sense of data, or *sensemaking*, is a cyclic process that involves cognitive activities for answering complex questions [5]. These activities involve repeated access to artifacts, identifying relevant information, finding information relationships, and presenting the answers in an understandable format [6]. Patients face a plethora of sensemaking challenges to manage their health. They need to assemble health information from different providers and identify outliers, correlations, and trends to become educated on health topics, drive decision-making, and formulate discussion points with health care providers [7]. Unfortunately, robust platforms to support patients in making sense of their clinical data are lacking.

Several commercial mobile apps such as Apple Health Records [8], iBlueButton [9], OneRecord [10], and 1upHealth [11], along with the academic web application Discovery [12], advance patient sensemaking by offering data visualizations and specialized views for data exploration. These views help patients uncover interesting patterns related to prevalence, periodicity, co-occurrence, and pre-post analysis of medical events. Typically, these solutions organize electronic health record (EHR) data by type, time stamp, or provider. However, such approaches provide very little support for patients in finding deeper connections between their medical records that are required for understanding health issues, reflecting on medical history, or preparing for clinical encounters.

Moreover, these apps do not allow patients to annotate their medical records or save their sensemaking progress, forcing them to remember findings or record them elsewhere. This necessitates patients to revisit the same data repeatedly to refresh inferences and recreate mental notes. Such work is typically tedious and frustrating, leading to anxiety and missing crucial information. Consequently, patients can form skewed health impressions, resulting in poor decisions or risky actions. In clinical visits, the inability to communicate the sensemaking insights to physicians may hinder optimal treatment, leading to repeated tests or medical errors.

To address these limitations, we explored an alternative solution that organizes EHR data into collections based on health issues and ongoing problems [13]. Inspired by findings that a problem-based view of EHR data improves clinician awareness, prioritization, and decision-making in the intensive care unit [14], we adapted a similar approach for patients anchored in the *data-frame* sensemaking theory [15]. Data frames (ie, structured mental models) or *collections* of health data, as we refer to them in our previous work [13], systematically break down problems and help in answering complex questions. These capabilities of the collections hold significant potential for managing health data effectively for all sorts of patients, particularly those with complex medical histories or multiple comorbidities [13]. By organizing abundant data around health issues, collections help patients avoid fragmented health impressions, a common challenge for those with multiple comorbidities. Patients who see multiple specialists can use collections to track the development of specific issues and share insights across providers, raising awareness and improving care coordination.

More precisely, our proposed concept of *collections* allows patients to dynamically organize, adapt, and explore their health information based on evolving needs and available data. For example, a patient managing cardiovascular issues might create a Blood Pressure collection to consolidate related records, which could later branch into more specific collections such as Extreme Blood Pressures or Blood Pressure Lab Work. These refined groupings help uncover patterns and dependencies among factors such as BMI, diet, cholesterol levels, and blood pressure, enhancing understanding and facilitating proactive management of health conditions. Gathering insights from the collections may also help patients have more productive discussions with their providers.

In our study, we extended the capacity to transform EHR data into collections and facilitated reasoning regarding them. As patients’ sensemaking of health data is driven by finding outliers, correlations, and trends [7,16], we enabled capabilities to identify data patterns within the collections. In addition, we supported the assembly of relevant medical records for the collections by helping patients visually explore, find temporal patterns [17-20], and make sense of their EHR data within a single, context-preserving view [12]. Acknowledging patient requests for automation [13], we offered manually assembled and system-assembled collections. We also allowed for personal data input through free-text notes, fulfilling previously identified patient needs [13].

A notable advancement in our work is moving from mock-ups [13] to a fully functional mobile app, Discovery. The app provides patients with a realistic platform to interact with collections, uncovering deeper insights into preferred mechanisms for creating, refining, and using these collections. Moreover, we intended to motivate patients to see collections as tools for self-advocacy during clinical visits, which was identified as a key use case in our earlier research [13].

### Objectives

In this study, we used the Discovery app to explore patients’ needs, preferences, and desired interactions for organizing EHR data and delve into potential use cases. More concretely, we asked the following research questions (RQs):

- What are the needs and feature preferences for organizing EHR data from multiple providers? (RQ 1)
  What are the patients’ experiences with creating and building collections?
  How effectively does our app allow patients to organize their EHR data using the concept of collections?
  How can we better meet patients’ needs for meaningful EHR data organization?
- What purpose would organizing the EHR data in collections have? (RQ 2)

To answer these RQs, we conducted a qualitative evaluation study with 14 participants.

## Methods

### Description of Discovery

#### General Overview

Discovery is a noncommercial iPhone app designed to help patients make sense of their EHR data. It introduces a novel concept that organizes EHR data into personalized, problem-based collections. In addition, it allows patients to add their own observations and insights, providing context and complementing their medical records with patient-generated data (PGD).

For this study, the app was restricted to accessing synthetic EHR data through a Fast Healthcare Interoperability Resources (FHIR) format [21] from the SMART Health IT repository [22]. Discovery accesses only structured EHR data and relies on a 2-level hierarchy. At the highest level, there are the record categories (“Conditions,” “Immunizations,” and “Vital Signs”). Each record category has multiple record types (“Vital Signs: Body Height,” “Body Weight,” and “Blood Pressure”), and each record type can have multiple instances (“Blood Pressure: systolic: 125, diastolic: 90,” and “date: 02/01/2022”). Each instance of data in our app is called a record and corresponds to an FHIR resource with standardized attributes.

#### Organizing Records in Collections

**Figure 1:**
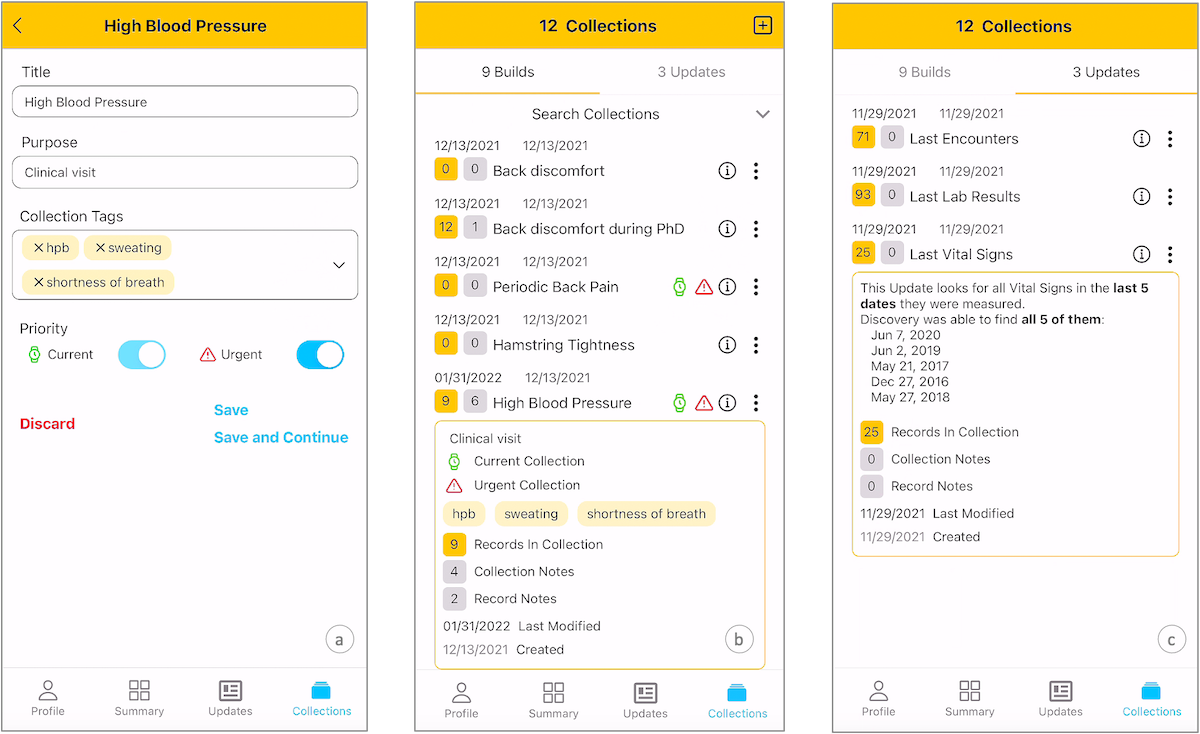
(A) Creation of a new collection; (B) list of patient-built collections—Builds; (C) list of system-generated collections—Updates.

The manually created collections are called Builds (creation shown in Figure 1A and list shown in Figure 1B), whereas the app-assembled collections are called Updates (Figure 1C).

Patients can manually create a new collection (Figure 1A) by entering a name and additional metadata. To prioritize and distinguish the collections, we introduced descriptors: purpose, tags, and priority. For example, in Figure 1A, the patient created a High Blood Pressure collection with the purpose Clinical Visit for an upcoming appointment. They also added tags such as hpb, sweating, and shortness of breath for reference. The priority descriptor indicates that the collection addresses a current and urgent issue. Patients can modify any of this information as the collection develops and changes.

As patients repeatedly create collections, their EHR data transform into a problem-based list, as shown in Figure 1B, which includes health issues such as back discomfort, hamstring tightness, and high blood pressure. Collections are displayed with their name, creation and last modification dates, record count, number of patient-added notes, and labels for current (green clock) and urgent (danger sign) issues. An information button provides a summary with explanations. For example, the High Blood Pressure collection was created on December 31, 2021, and last modified on January 31, 2022. An information panel summarizes its contents: 9 records, 6 notes (4 for the collection and 2 for individual records), and tags for detailed search (hpb, sweating, and shortness of breath).

Updates automatically familiarize patients with the latest relevant events without requiring any action on their part (Figure 1C). The app scans all records and matches predefined templates, such as recent encounters, laboratory test results, and vital signs. In Figure 1C, 3 Updates are listed—Last Encounters, Last Lab Results, and Last Vital Signs—based on the last 5 dates when corresponding records were logged by the provider. The list entry follows the same structure as that of the Builds without the labels for currency and urgency. Although records in an Update cannot be changed, patients can add and remove notes. Patients can also clone an Update into a new Build and rename it if needed, allowing them to reuse and customize the assembled records for specific purposes. The reason for having this distinction between Updates and Builds is to delineate what the system and the user have produced and which entity is responsible for the collection that might have led to certain actions.

#### Identifying Relevant Records and Patterns for Collections

**Figure 2:**
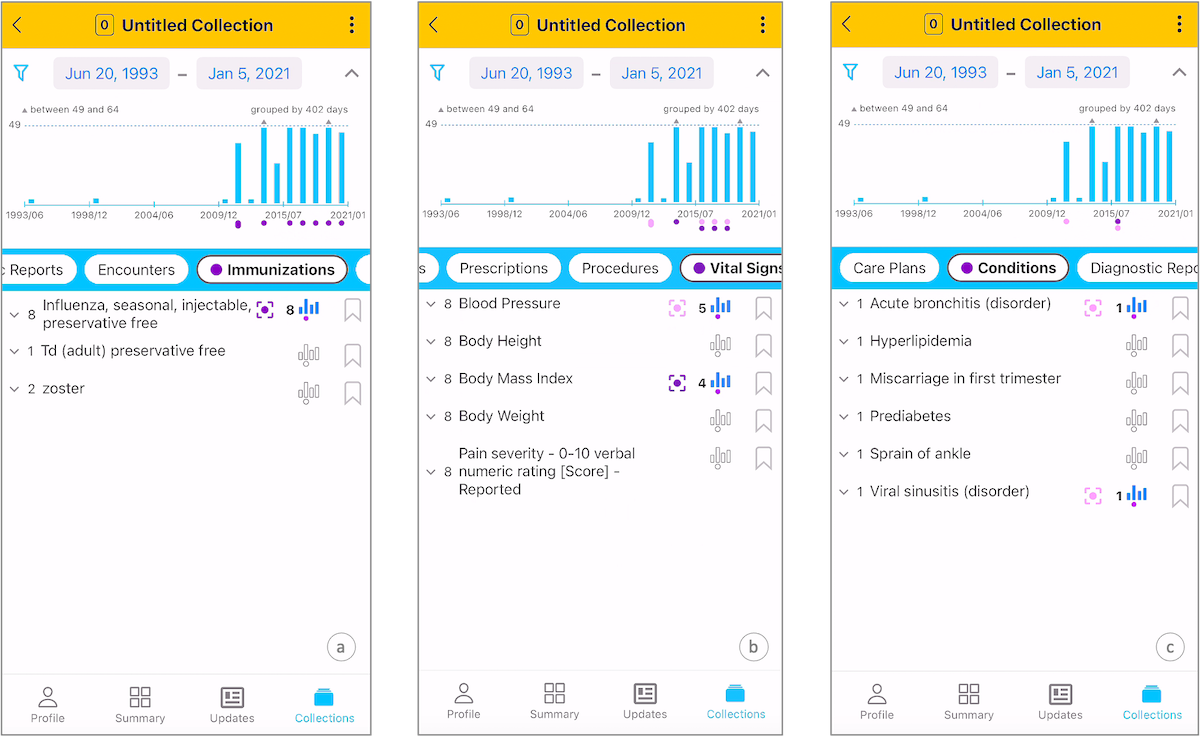
Finding medical event patterns: (A) periodicity of influenza shots (showing when and how frequently influenza shots were administered), (B) co-occurrence of high blood pressure and high BMI, and (C) absence of respiratory conditions after an amoxicillin prescription.

Discovery offers an interactive visualization to find patterns within records. The Timeline depicts record counts in equal time intervals, showing the prevalence of medical events. A dotted horizontal line marks the threshold above which the volume of records is considered abnormal, corresponding to the mean record count per interval. Gray triangle glyphs indicate values between the mean and 2 SDs, whereas red triangles highlight values of >2 SDs. By highlighting individual records or entire record types, patients can explore patterns that may be saved in existing collections or trigger the creation of new collections. For example, Figure 2A shows periodicity of influenza shots (when and how frequently influenza shots were administered), Figure 2B shows the co-occurrence of high blood pressure and high BMI, and Figure 2C shows the absence of respiratory conditions after an amoxicillin prescription.

The FHIR resources (ie, medical records) are represented with Record Cards, which display the clinical information in human-readable format. Patients can use a Filter and Date Picker to narrow down record categories and time frames for displayed records. Selected record categories appear in a Sliding Tabs control, allowing patients to swipe left or right for immediate access. Clicking on a record category in the Sliding Tabs organizes it by record type, represented with Accordions (eg, the Accordion for Immunizations category will have 3 sections for the Flu shot, Tdap, and Zooster record types). Patients can expand the Accordion sections to scroll through individual Record Cards. For example, Figure 2A shows 8 Record Cards for the influenza shot under the Immunization Accordion section. Accessing and revisiting records involves swiping the Sliding Tabs and selecting record categories, with Accordions retaining their expanded or collapsed state and scrolling position. This method is more context preserving compared to existing solutions, which require repeated back-and-forth navigation through different views for each record category and record type.

#### Producing Insights for the Collection

**Figure 3:**
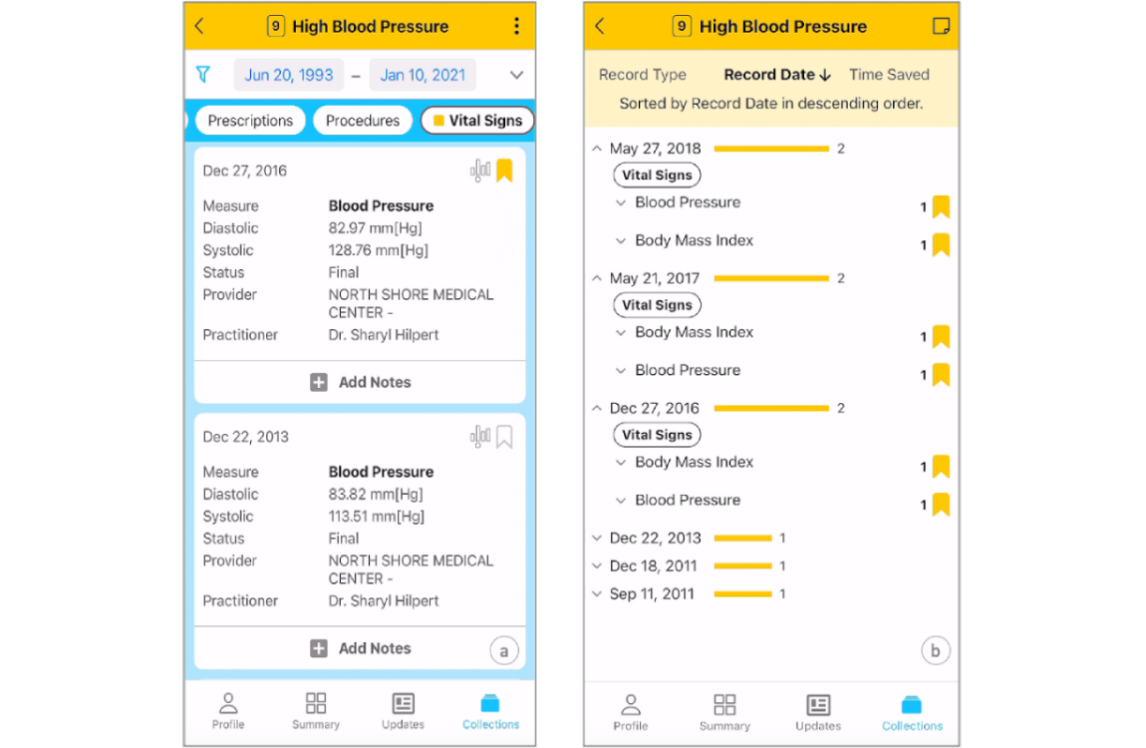
(A) Saving a record to a collection and (B) reviewing the collection and inspecting data patterns.

Patients can save individual records or entire record types by tapping the bookmark icon in the corresponding Record Card or Accordion section. Figure 3A demonstrates adding individual Blood Pressure records to a collection using a Record Card. Records can be removed from collections by tapping the selected bookmark again.

To identify data relationships and produce insights, patients can inspect a collection in the Collection Review (Figure 3B). Here, records can be viewed by type, date recorded in the provider’s EHR system, or time added to the collection. When sequence matters, records can be ordered chronologically. Patients can also remove records in the Collection Review by tapping the selected bookmark. For example, in Figure 3B, the patient views records grouped by recording dates in descending order, with yellow bars indicating record counts by date. They observe high blood pressure and high BMI co-occurring 3 times, suggesting a pattern that may need further investigation or discussion with a physician.

#### Supporting PGD

**Figure 4:**
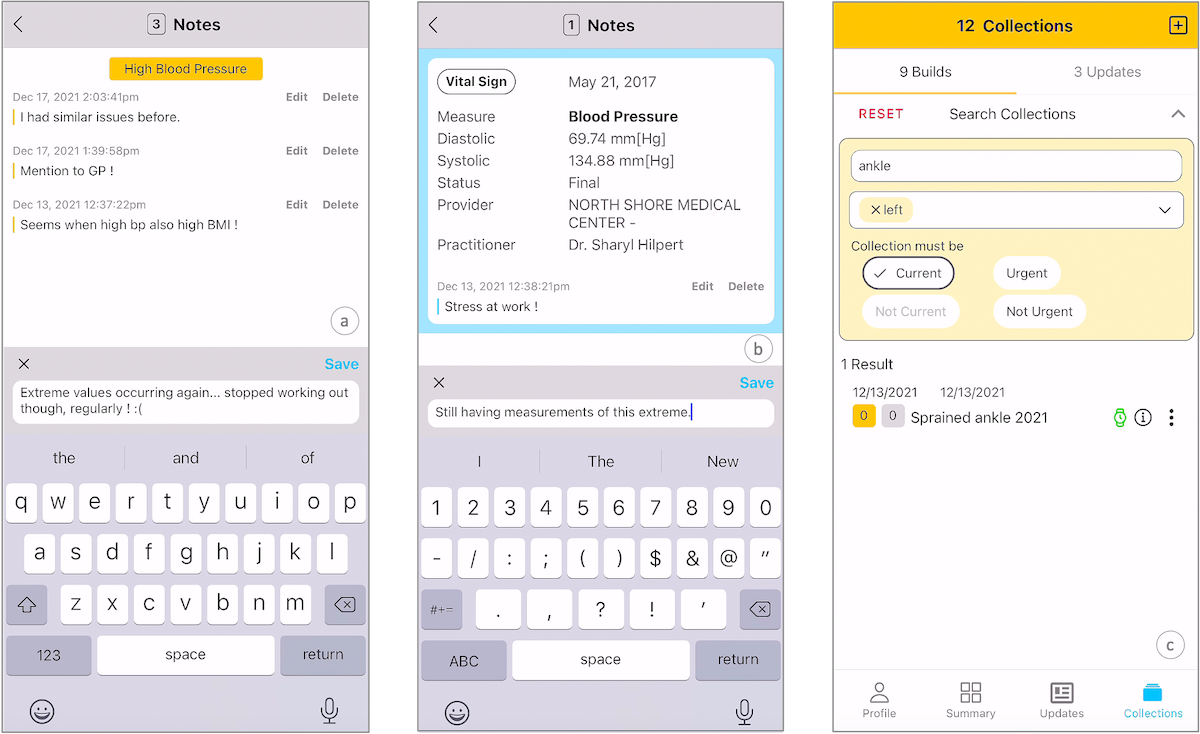
(A) Adding a note to a collection, (B) adding a note to a record, and (C) searching the collections.

Patients can enter notes for a collection (Figure 4A) or individual records within it (Figure 4B) to add personal insights, progress, observations, details, or disease journal entries. Notes can be modified or removed at any time. In Figure 4A, the first note provides context on past high blood pressure experiences. The second note serves as a reminder to mention occasional high blood pressure to the general practitioner. The currently created note adds context about noticing changes in blood pressure after stopping regular workouts. In Figure 4B, the patient contextualizes a high blood pressure measurement taken during a stressful period at work and notes the recurrence of high values.

#### Searching the Collections

Free-text search targets the collection name, purpose, and notes (Figure 4C), as well as tags and priority. Results dynamically update as the search query is constructed.

### Study Design

#### Participants

We recruited 14 participants from our email list compiled from previous recruiting efforts and Craigslist. This number is sufficient to uncover usability issues and provide rich findings as per current design research practices [23] and literature on user feedback quality [24]. We balanced the sample by age, gender, and medical history (including healthy individuals, those with acute episodes, and those with chronic illnesses). Eligibility criteria included adults fluent in English; possessing an iPhone (iPhone 6 or above) and a laptop or desktop computer (screen size of 13“ or more) with a stable, fast internet connection for both devices; and with normal or corrected vision, no color blindness, and medical records from one or more providers. Medical history was self-reported.

Table 1 illustrates the detailed participant demographics collected using the questionnaire from Table S1 in Multimedia Appendix 1. Our 14 adult study participants included 10 (71%) women and 4 (29%) men aged 24 to 61 years (mean age 35.6, SD 12.6; median 30.5 years). All had some college experience: half (7/14, 50%) held bachelor’s degrees, 14% (2/14) had some graduate experience, and 14% (2/14) had completed master’s degrees. Participants had between 2 and 15 health care providers, with half (7/14, 50%) having ≥6. The 29% (4/14) of the participants who were healthy saw physicians a few times a year. Those with chronic illnesses had been managing their diseases for 1 to 20 years.

All participants were comfortable with daily technology use, and 21% (3/14) had work experience in data analytics. Most (11/14, 79%) used third-party apps to track mental health, weight loss, sleep, and exercise. All but 1 (13/14, 93%) used provider-patient portals to review test and laboratory results, refill prescriptions, and schedule appointments. However, participants found it cumbersome to remember multiple passwords and difficult to find specific information due to interface issues. Data sharing among providers was often slow or impossible, forcing participants to print and assemble records for clinical visits.

**Table 1:** Participant demographics.

Participant ID	Age (y)	Sex	Educational level	Medical issues	Health care providers, N
P1	61	Male	2-year college	High blood pressure, sleep apnea, fatty liver (stage 3), and liver transplant	7-8
P2	45	Female	Some college	High blood pressure, prediabetes, obesity, and hypothyroidism	6-7
P3	51	Male	Some graduate school (incomplete degree)	Hypertension and gout	2
P4	24	Male	Bachelor’s degree	Childhood asthma, recent hernia surgery, and mental health therapy	5
P5	24	Female	Bachelor’s degree	No chronic issues	2
P6	27	Female	Bachelor’s degree	No chronic issues	3-4
P7	28	Male	Graduate degree	Physical and mental (20 years)	Approximately 13
P8	37	Female	Master’s degree	Thyroid condition (many years)	3-5
P9	29	Female	3 years (incomplete degree)	Chronic migraines (10 years) and hemiplegic migraines (1 year)	≥12
P10	59	Female	Bachelor’s degree	Yes—5 years	10-15
P11	25	Female	Bachelor’s degree	No chronic issues	3-5
P12	33	Female	Master’s degree	Anemia (2 years) and IBS^a^ (10 years)	3
P13	19	Female	Some college (incomplete degree)	Asthma (since she was a baby) and EDD^b^ (10 years)	>5
P14	32	Female	Master’s degree	No chronic issues	3

^a^IBS: irritable bowel syndrome.

^b^EDD: eosinophilic digestive disease.

#### Procedures

**Figure 5:**
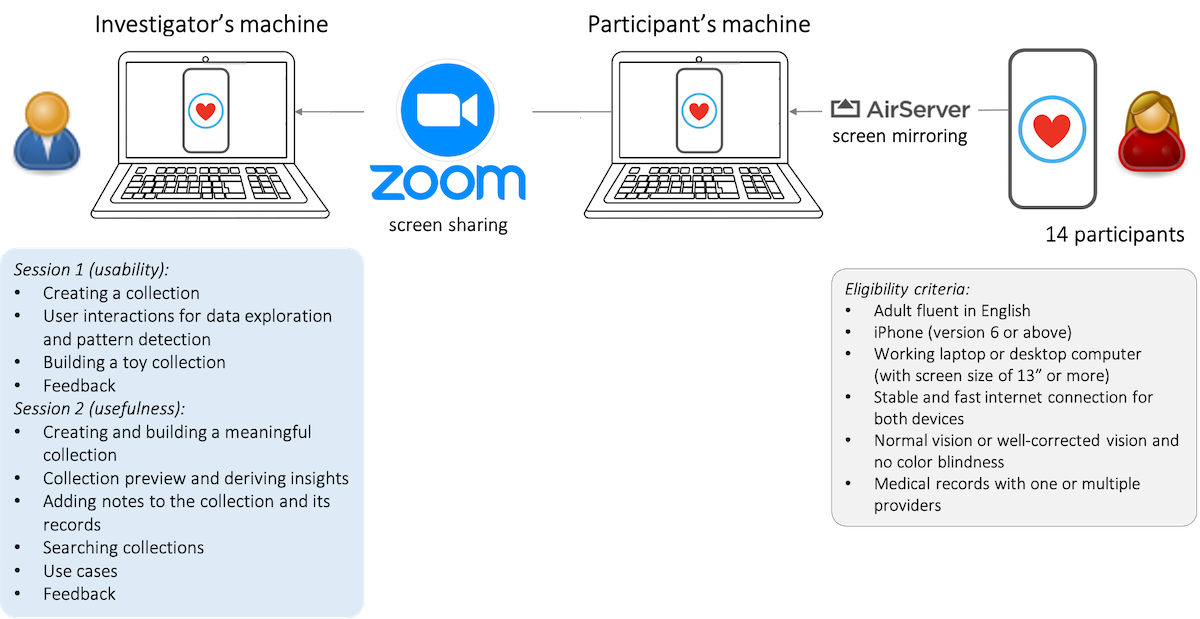
Study design—14 participants for 2 one-on-one sessions with a researcher. Session 1 focused on usability, and session 2 examined usefulness. Feedback was collected on how participants would use collections for their own data and needs.

Inspired by existing patient portal usability studies, the study procedures were tailored to our RQs [23,25,26] and are presented in Figure 5. The study was conducted remotely via Zoom (Zoom Video Communications) meetings. Participants downloaded our app on their iPhone using Apple TestFlight for beta testing [27] and mirrored their iPhone screen on their laptop using AirServer (App Dynamic ehf.) [28]. The laptop screen was then shared with the researcher for observation.

Participants attended an initial 60-minute session (session 1) and a follow-up 45-minute session (session 2). Both sessions used the concurrent think-aloud protocol to gather rich qualitative data on user needs, perceptions, and preferences efficiently [29]. Textbox 1 illustrates 2 example topics and detailed tasks for creating a toy collection and realistic collection from sessions 1 and 2, respectively. The full set of 13 topics for both sessions and their detailed tasks that follow a similar structure are shown in Tables S2 and S3 in Multimedia Appendix 1.

In session 1, the researcher first collected demographics and digital health consumer information from participants (Table S1 in Multimedia Appendix 1). The session then evaluated the usability of the app (RQ 1). Participants were introduced to the app’s key concepts and features and were then given tasks to learn the interactions for creating and assembling relevant records in collections. After each task block, participants provided feedback. Tasks included creating a toy collection, adding descriptors, exploring scoping mechanisms for record categories and time ranges, navigating records using the Sliding Tabs, and inspecting the interactive Timeline visualization. Participants also completed tasks related to finding periodicity, co-occurrence, and pretest-posttest analysis of medical events using the Timeline; saving records to a collection; and reviewing it. The session concluded with feedback on the intuitiveness, usability, and usefulness of creating, building, and reviewing collections, as well as data exploration and pattern detection features (Textbox 2).

Session 2 focused on the usefulness of collections in real-life settings (RQ 2). Participants performed tasks involving creating a collection tied to a specific issue (eg, high blood pressure) and purpose (eg, clinical visit). After each task block, participants provided feedback. Tasks included creating a High Blood Pressure collection, adding descriptors for a clinical visit, selecting records related to high blood pressure, identifying patterns in selected records, adding notes to the collection and specific records for clinical context, and using the search feature to find other collections. The session concluded with feedback on the usefulness of collections, brainstorming real-life use case scenarios, and suggesting potential improvements (Textbox 2).

**Example tasks from sessions 1 and 2.**

**Session 1: creating a collection (5 min)**

- For the purposes of learning the basic mechanics around building a Collection from scratch, first Create a new Collection and name it “Toy Collection.”
- Now, let’s add some descriptors about the Collection.
  Please add a purpose to the Collection, something related to learning about this app.
  Now, add a couple of tags to further describe, summarize or annotate the Collection for future quick access.
  Finally, specify the priority of this Collection by marking it as urgent.
- What was your experience with creating the new Collection?
  How intuitive was it? Have you seen similar interactions elsewhere?
  How useful do you find the descriptors for the Collection?
- Now, let’s go back to the list of Collections. Let me know how can you see the details about the Collection you just created?
  How intuitive was it?
- What are possible improvements?

**Session 2: building a collection (15 min)**

- You will now create a Collection that is more realistic and meaningful for use in a real-life scenario. We will assume that you are preparing for an upcoming visit to your physician’s office related to potential issues with high blood pressure. Create a collection called “High Blood Pressure.”
  Add the purpose for the Collection
  Add a few tags
  Mark its priority
- Add the Records with blood pressure with systolic value over 120 and BMI over 30.
- What was your experience with assembling the Records for the Collection?
  How laborious was it?
  What are some ways in which we can make this assembling process more efficient?
  How do you feel about having the system prepopulate the Collection for you and let you modify it afterwards?

**Feedback collected at the end of the study sessions.**

**Session 1 example tasks**

- What was your impression of this app?
  What did you like?
  What did you dislike?
- How intuitive was the app?
- How easy or hard was it to explore the data?
- How useful were the features in the app to identify patterns in the data?
- How did you like the mechanism for saving Records in the Collection?
- What are some improvements you would like to see?

**Session 2 example tasks**

- How useful do you think the Collections can be for you?
- What are some use cases for the Collections that you can think of?
- What are some improvements you would like to see for the Collections?
  Automatic support for building Collections?
  Automatically finding data patterns in the Collections?
  Patient-generated data?

#### Data Collection

We recorded the Zoom meetings for audio and video capture of the entire interaction. The first author also took notes during the meetings.

### Data Analysis

Audio recordings were transcribed using Rev (Rev.com, Inc). We analyzed video recordings for additional context and a deeper understanding of participant comments during the think-aloud protocol. Video annotations were added to the transcripts and session notes [23] for reflexive thematic analysis [30]. The first author began by open coding the textual data. Emerging categories were reconciled in meetings with the second and last authors to identify use cases and detailed approaches to organizing and annotating EHR data. These themes were validated in a group meeting with researchers unfamiliar with the collection concept and with our app.

### Ethical Considerations

The Harvard Faculty of Medicine Institutional Review Board approved this study (protocol IRB20-1757). Participants signed a consent form, which also allowed them to opt out of the study at any point. Each participant received a US $40 Amazon gift card as compensation. The data obtained from the study sessions did not include any identifiable information about the participants and were stored on a password-protected computer with encryption. Only the research team had access to these data.

## Results

### Overview

The results from our study are organized and analyzed around five qualitative themes, two applicable to RQ 2 and three applicable to RQ 1: (1) using collections for personal benefit (RQ 2), (2) using collections in a clinical setting (RQ 2), (3) creating and building collections (RQ 1), (4) enhancing collections (RQ 1), and (5) accessing collections (RQ 1). In the remainder of the Results section, we characterize the participants and report on these themes using 16 quotes from 11 different participants. The participants are labeled as P1 to P14.

### Purposes for Organizing the EHR Data in Collections

#### Using Collections for Personal Benefit

##### Quick Access to Information

Participants perceived the Collections feature as a way to index information at a suitable level of granularity for health issues, topics of interest, or conditions to monitor. They expected that this would give them quick access to current or urgent problems that were being managed:

Personally I would like to monitor my asthma because I am using medication for that, the typical inhaler, but I would like to monitor, these days I had certain attacks or shortness of breath, so collections, having it back for that specific condition is very useful to me, because I wouldn’t have to speculate about when my last attack was or when my last appointment date was, it’s right here for me to access.

P13

##### Reflection on Medical History

Participants viewed collections as a tool to construct and understand their medical history. They expected collections to serve as a repository for the health issues they faced, aiding in reflecting on the existence, prevalence, and development of their health conditions:

This will be able to find what happens when I have, in my case, headaches related to my high blood pressure and nothing else, or if you’re sick for the flu, influenza and you have fever, it’s just related to the influenza, not to the COVID.

P1

##### Keeping Track of Health Status

Most participants envisioned using collections to track their health status proactively. This included monitoring urgent issues needing immediate attention, unstable conditions requiring frequent observation, and treatments needing careful monitoring. Participants also wanted collections to track abnormal laboratory test results and vital sign values across various health issues:

Well, for me, it’s kind of good [to have medical records organized in collections], especially, for example, blood samples, especially those with high triglycerides or something. Maybe I can collect them and see whether there’s a trend for this month, or for January, I’m high in this one. Then second month, I’m also high, so maybe I can lower it down...For collections, just categorize those. Which are high, which are low.

P5

##### Journaling Daily Events

Most of the participants envisioned using collections as a personal diary for coping with diseases and logging measurements and their effects on lifestyle. They wanted to track challenges, successes, and progress toward finding solutions and monitor disease developments:

What I’ve done is I’ve taken all of my videos and stuff since February. Like I said, I’ve been to eight different doctors and I’ve shown them, this is the progress of what’s happened from...I’ve had two surgeries before this surgery where they lanced it, cut it, drained it. Nothing happened. The cyst came back and then it went into my bone. So I’m able to bring these photos, I’m able to bring this timeline, I’m able to bring my frustrations and show this doctor within 30 seconds [using a collection], look, this is what it looked like. And this is my own diary, my own history. It’s very important because they’re a doctor. They don’t know me, they don’t know what it looked like on day one.

P10

##### Learning From Past Experiences

Nearly all participants wanted to use collections to identify trends and patterns in their ongoing health experiences, including co-occurring symptoms and treatment effects. They also intended to log food intake, sleep, activity, or stress factors to find triggers for symptoms:

I think I could definitely use them there. It’s a lot easier now because I could highlight the certain event, and put like my triggers down with it, like migraine on the third was this, you could even put what you took with it. So for me, I would look back and be like, “Oh, I can tell my doctor that, I’ve had 10 migraines. I took a medication with these three. What’s my options.” So I think that would be great, it’s a great tool that I can actually do that with this app.

P9

#### Using Collections in a Clinical Setting

##### Preparation for the Clinical Visit

Most participants would use collections to prepare talking points for clinical visits combining personal measurements and notes with medical records from other providers (eg, laboratory and test results). This was crucial because their physicians often did not have access to external data:

For collections, I would say that if I’m meeting with multiple providers about one health issue, I could see myself combining all my records there so that multiple providers can see each other’s records...I would probably [use the notes], if I needed to jot down a certain time that I took a measurement, or if my doctor told me keep track of what you ate that day, or kind of anything that I would want to have the details for the next time that I go and see the provider.

P6

##### Relying on Collections During Clinical Visits

All participants wanted to share collections with their physicians during clinical visits to establish ground truths, raise awareness of other providers’ information, and provide transparent talking points. They believed that adding PGD to collections could describe what happened between visits and raise their physicians’ awareness:

If you have a condition, you need to check on your blood pressure. You need to communicate that with your doctor, so you can add a note [in the collection] saying like, “Latest, highest blood pressure from this week,” from a date.

P14

In addition, most participants felt that taking in-visit notes and saving them in a collection could help them understand care plans and take appropriate actions afterward:

Maybe [taking notes in the collection for the clinical visit] just for your own personal reference or if you wanted to bring it up later on in another appointment or something, or just maybe, I guess, just general recording of something that happened during that visit.

P11

All participants saw physicians as essential partners in reviewing collections and deciding on actions based on their contents. This was primarily because most participants doubted their expertise in determining what should go into collections or which collections to create. While they were very open to include their physicians in the collection curation, some feared that they might overburden physicians with verification inquiries:

I guess I would definitely do that [look for patterns in the data and store them in a collection] just because I can actually consult the doctor. Is this actually correlated or will I have to change my diet because it affects this? At least you can ask the doctor, or confirm whether that is true or not.

P5

### Needs and Feature Preferences for Organizing EHR Data in Collections

#### Creating and Building Collections

##### Manual Workflow for Creating and Building Collections

Overall, all participants expressed satisfaction with the clarity and simplicity of the mechanics to create collections and save records in them. However, those with less medical knowledge and disease experience (relatively healthy and recently diagnosed individuals) thought that initiating and building collections was challenging. For them, it was not always clear what issues deserved separate collections, what records to include in a collection due to delicate dependencies, and why they should invest substantial time and effort in assembling collections. In contrast, the more experienced (chronic) patients were relatively confident in their ability to carve a personalized view of their EHR data. However, some did acknowledge that they might not be as exhaustive and reliable in their data organization.

Participants quickly mastered using the Sliding Tabs with Accordions and appreciated the context-preserving record exploration. However, most of the participants found it challenging to assess the relevance of individual records due to unexplained clinical language and attribute values. Participants requested explanations, prominent visual cues for abnormal values to attract their attention, and time-series visualizations for additional context and noticing trends. They also wanted to be able to sort by date or attribute value, with filtering capabilities that also included adding the filtered records in bulk to a collection.

Some participants desired collections with richer internal structures, recognizing the need to identify subsets of records and their importance within a collection. They suggested linking groups or individual records using specific annotations for easy identification during visual inspection or search:

...maybe if certain records are related to each other. So I would want to mark that. And then maybe just have a way of sorting down based on certain labels.

P8

##### Automatic Support for Building Collections

Participants were highly receptive to discussing ideas that could automate building collections. They were interested in seeing their records automatically put into collections based on provenance (provider, physician, hospital, location, and date) and clinical meaning (condition, disease, organ, organ system, and abnormality of the values across records). Some also suggested grouping records into collections based on personal annotations. For all these groupings, they wished to be able to edit the collection manually:

If I had neurological problems, neurology collection. If I had urological problems, urology collection. I think that for me at least would seem a more straightforward way to categorize them. But from the categories I’ve already seen, I find those useful.

P4

Several participants suggested using a “seed” to automate collection building, such as naming a collection, adding keywords, or including a few records:

And I think a lot of patients don’t know where to start, what data to begin with. So if it’s something that’s already preset, they say, “Okay, I’m suffering from depression or I have diabetes.” And the system pulls the different data points that they would need to look at for someone who’s diabetic or someone who’s dealing with depression, I think that’s helpful. Because sometimes, the problem is you don’t know where to start and you don’t know what to look for.

P11

Finally, most of the participants wanted to receive automated help to add or remove records for a collection that had already been created. Few described wanting to choose from a list of suggested records based on the existing content of a collection, revealing records and record patterns otherwise invisible to them. Others saw this automated record offering more as an idea generation approach—needing some follow-up validation, including taking it up with their physicians:

When I work, I want to listen to some music and then I’m like, okay. I just don’t know what’s next. I need something similar to this, the same vibe, but I just can’t think about that. And then there is suggestions. And yes, some of it’s weird. But maybe like the doctor can also have some help here, and when you review the collections together, they might say, “Hey, listen, this is what the system gave you and that’s great. Let’s remove a few things. I would suggest you add a couple others. And whatever you put there, it’s also fine. And let’s keep it that way.”

P6

#### Enhancing Collections Through Personally Provided Data

##### Making Collections Complete

Participants strongly expressed the need to complement their EHR data with daily entries from sensors; self-monitoring devices; and manual measurements of symptoms, treatments, and outcomes in various formats (text, photos, videos, and scanned documents):

Actually, I think you can sort of restructure the whole core of the collections on top of two main pillars. The first one would be all of the doctor’s data, which is basically hard data, which allows you to diagnose, allows you to run statistical analysis...That could be part of the core data, but all of the context, maybe I’m getting this shortness of breath in my home, watching my TV, might be added by the notes. You have these two types of data. By adding the user data, would allow me to get context, give context, which is important and will allow me to, on a daily basis, keep a record, which in case of data like shortness of breath, I’m having, I’m not having. Would allow the doctor to have a really unbiased input on symptoms I’m having.

P7

Participants wanted to log detailed observations and measurements, pairing treatments with outcomes and symptoms with triggers. They suggested dedicating a special PGD record category for these data, with some preferring complex structures and others favoring simple data entry options:

I would probably use the notes quite often just to maybe outline the symptoms I was experiencing and the steps I took to alleviate those symptoms or which doctors I contacted.

P13

##### Making Collections Distinguishable

Participants liked the existing collection descriptors and suggested additional ones. They wanted labels for clarity (clear, unclear, or potential issue), stability (stable or unstable), progress toward resolution, development stages (nonthreatening or threatening), and a list of involved providers and physicians. When collections were related to clinical visits, participants wanted to specify the targeted physicians.

##### Making Collections Actionable

Participants believed that organizing medical records by health issues in collections was a good start but thought that actionability could be improved with specific insight notes and annotations applied to entire collections:

And then as far as the purpose of adding a note to the whole category, I would say that, like you said, if you happen to notice any patterns when you’re looking at the data, or basically I would use it for any general or bigger-picture takeaway that I wanted to tell my doctor, “Hey, I noticed this” or something and I wanted to bring it to their attention.

P6

Participants envisioned using collection-wide notes to summarize contents or purpose, track progress, describe issue development, and highlight special events. They also wanted notes representing care plans and actions prioritized in a to-do list. Participants intended to use collections to prepare for clinical visits with questions, reminders, and critical measurements. They also saw value in adding collection notes about visit outcomes, key takeaways, and next steps.

Some participants wanted to annotate and highlight keywords or add tags to free-text notes for organized review and pattern identification.

#### Accessing the Collections

All participants emphasized the importance of fast, reliable access to collections and their contents. They primarily relied on collection descriptors but also desired a deep search feature that would scan through individual records, notes, and annotations within collections.

## Discussion

### Principal Findings

We identified 3 principal findings of our study. First, participants embraced the collection concept. Unrestrictedly organizing EHR data into collections that map medical records to health issues and track ongoing concerns gave participants a sense of ownership. They felt empowered by developing personalized health narratives that could aid in self-management and communication with their physicians, enhancing their self-advocacy. Second, while participants easily mastered the interface for initiating and adding records to collections, they found the process laborious. They lacked confidence in selecting appropriate records due to limited medical knowledge and requested additional visual cues, explanations, and automatic collection features. There was concern about potential self-misguidance without physician verification. Third, collections would need richer PGD capabilities for adding contextual information not found in participants’ EHR data, logging observations, and labeling data. This would enhance the comprehensiveness and accuracy of their health narratives and support foraging, sensemaking, and action taking.

### Interpretation of the Findings and Contributions

#### Overview

On a broader scale, this work contributes to patient-centered care. This is achieved by demonstrating potential to enhance patients’ grasp of their health, encourage self-advocacy, and improve patient-provider communication. More accurately, there are several concrete contributions of our work that can be considered as proxies toward achieving the aforementioned objectives: (1) encouraging patient ownership of their EHR data by organizing them into personalized, health issue–based collections; (2) understanding patients’ perceptions and preferences for creating, building, and using these collections; and (3) offering design insights for automating collections, integrating rich PGD, enhancing access to collection contents, and using collections to facilitate patient-provider communication.

Going forward, we will situate our findings within a sensemaking framework and discuss contributions related to 3 key patient needs: increasing awareness through independent health sensemaking, proactivity through efficient action taking, and self-advocacy through incorporating evidence-supported patient perspectives into patient-provider communication. We will elaborate on how collections can meet these needs and offer design implications to enhance their capabilities.

#### The Role of the Collections in Supporting Sensemaking

To explain the collections’ role in sensemaking, we used the model by Pirolli and Card [6], which divides the sensemaking process into 2 subcycles: the foraging loop and the sensemaking loop. On the basis of this model, collections can be described as a space for assembling relevant data about a topic, finding relationships between them, and storing outcomes from the sensemaking. In the foraging loop, patients gather relevant records to answer questions such as the following—“Is there a relationship between my weight and blood pressure?”—and save them in a collection, such as Weight vs. Blood Pressure. In the sensemaking loop, patients identify information relationships within the collection that they capture in notes, such as instances where there was co-occurrence of high blood pressure and high body mass. These notes help argue hypotheses such as the following: “My blood pressure is high when I’m overweight.” The outcome of this sensemaking process could be a comprehensive note for a clinical visit.

While, in their current form, collections respond to the needs of the sensemaking model by Pirolli and Card [6], improvements can be made to make this more efficient. This study revealed that medical records alone are not enough for reliable sensemaking. Adding PGD such as symptoms, measurements, outcomes, and everyday events is essential for creating comprehensive collections. The foraging loop can be made less laborious and time-consuming if there are additional visual cues, medical explanations, filtering capabilities, and automatic support to improve the relevance and reduce the effort of assembling collections. The sensemaking loop could be improved by adding more schematization capabilities such as annotating medical records and PGD to identify patterns later (eg, symptom triggers, medication effects, and correlations) and grouping records within collections, labeling those groups, and establishing group relationships with explanations (eg, linking “cholesterol lab results” with “food intake” as “food effects on cholesterol” in a High Blood Pressure collection).

#### Reliability of Collections

According to our study, there are 4 main factors that can influence the reliability of the collections: robust coverage of health issues, provision of PGD, grouping and linking of medical records and PGD within a collection, and verifying the contents of the collections. This reliability is related to collections’ capability to aid in creating personalized but realistic health issue narratives, support self-management, and stimulate awareness and proactivity.

##### Robust Coverage of Health Issues: Relevance Assessment

Collections should ensure that patients can create collections for their most important health issues to support awareness and proactivity. Participants desired visual cues, explanations, and automatic support to determine which collections to create and what records to include.

While participants found the context-capturing data exploration using Sliding Tabs and Timeline convenient, they needed more to identify relevant records quickly. Future tools could incorporate Accordions that summarize record types, graph values over time, and highlight abnormal or extreme values. In addition, patients should be able to expand individual records to see explanations of clinical terms and clinical meaning interpretations. While there is existing work related to visualizing time series of EHR data [17-20] and automatic provision of short explanations [31], this study shows the need for combining them in a new way to support relevance assessment for a novel purpose—constructing collections. Finally, patients should be able to order and filter records by attribute for quicker browsing and bulk addition to a collection.

Automatic support should also be provided for creating and building collections to save time and ensure robust, reliable coverage of health issues. Our previous work highlighted the need for automating collections [13], and this study highlights a clear preference for automatically grouping medical records by clinical meaning—whether *thematically* or *guided by patient input*. Thematic collections would be those that tie records together based on conditions (bronchitis or diabetes) and procedures (stent placement or appendix removal) or with respect to organs (heart or kidneys) or organ systems (cardiovascular or renal). Alternatively, patients could specify a seed by setting parameters such as the collection’s name, tags, or initial records. The system can offer candidate records to include or delete with confidence scores and explanations. Patients could then refine these system-generated collections by adding or removing records, PGD, and tags.

Addressing automatic support for collections may be challenging due to subtle relationships between medical records [32-34]. However, starting with easier constructs such as *time stamps* (eg, medical records from the same day, week, or month), *FHIR links* (eg, medical records from the same encounter or physician), *abnormal values* based on well-established clinical guidelines (eg, high or low blood pressure or cholesterol), *test findings* (eg, positive and negative), and *patient tags* (eg, triggers or pivotal events) is a feasible approach.

##### Provision of PGD: Sensemaking Data Completeness

Collections should include PGD to improve data completeness for sensemaking. Previous research has suggested that maintaining consistent PGD logging over time is difficult [35]. However, this should not be considered a barrier or a prerequisite for the collections’ success. Patients’ motivation and preferences for PGD logging intensity vary based on their disease self-management state [36]. When setting goals and learning strategies, patients prefer meticulous data collection. Once goals and strategies are in place, logging intensity decreases. In addition, if physicians require PGD logging for treatment planning, patients are motivated to engage in it [36,37].

Thus, collections should enable flexible and efficient PGD logging. Disease-specific contexts such as irritable bowel syndrome [38], diabetes [7], and migraine [39] have explored health sensemaking without focusing on diverse data types. This contrasts with patients’ desire to log PGD for various medical issues within a single application [40,41] using a universal logging model for different observations [42,43]. To address this issue, we propose a straightforward workflow where patients initiate free-text entries and use tags to specify the type, quality, or other details. This mechanism allows for quick data capture and embellishment at convenient times.

Tags can classify PGD as *clinical observations*, *everyday life events*, or *notes*. Further specification can be added using tags such as *symptom*, *measurement*, *treatment*, and *outcome* for observations; *meal*, *exercise*, *meeting*, and *deadline* for life events; and *context*, *personal note*, and *visit note* for notes. Additional tags such as *absent*, *normal*, *high*, *low*, *extreme*, *improvement*, *deterioration*, *pivotal event*, *trigger*, or *relaxer* can be used for further detail that captures the quality and importance of the logged data. In addition to these system-offered tags, patients can also create their own custom tags for better personalization.

##### Grouping and Linking Within Collections: Schematization Capabilities

Patients need to connect medical records and PGD within collections for easier sensemaking. Future tools should add structure by enabling record grouping and linking of groups or individual records. This helps highlight important subsets of records and trace major conclusions as collections grow.

We recommend using a simple yet powerful tagging concept. Records sharing the same tag can form a group, whereas links between groups can be specified using related tags. The same mechanism can link individual records with other records or groups, providing nuanced sensemaking. This approach aligns well with the proposed PGD tagging model that can be applied to medical records as well.

##### Collection Verification

Collections represent the patient’s personalized perception of their health and issues. As such, they should undergo occasional verification by the patient’s physician for safe decision-making and action taking. While collection verification may add to the physician’s workload, it can inspire and enable patients to manage health issues more independently. That said, patients should consider the physician’s workload before requesting verification [44].

Future tools could allow for the conversion of a collection into a well–laid-out PDF document capturing all its contents. This document can be printed for review during a clinical visit or shared as a PDF attachment in the patient portal for verification at the physician’s convenience.

#### Taking Actions Based on Collections

While annotating PGD is known to aid learning and disease self-management [7,45], our findings reveal that annotations can also enhance EHR data, creating synergy with PGD. Participants expressed a desire to annotate their data for various purposes: identifying triggers to avoid or encourage certain behaviors, marking pivotal events to remind them of shifts in health attitudes and management, and labeling outcomes as desired or undesired to evaluate treatments and strategies. These capabilities can be easily implemented using the previously elaborated tag-based design for linking records.

To increase the awareness and prioritization of collections, we previously proposed *collection descriptors* such as topic, urgency, currency, and sentiment [13]. Participants found value in these descriptors but expressed a need for additional ones that can be classified as *patient specified* and *data driven*. Future tools may include patient-specified descriptors for *clarity* (eg, is the diagnosis clear?), *stability* (eg, is the treatment working consistently?), *severity* (eg, is there a significant medical risk?), and *progress markers* (eg, is the issue substantially resolved?). Data-driven descriptors could be derived from the collection data, indicating the *time span* (from the oldest to the latest record) or listing the *physicians involved* (the providers and physicians the records came from). Both types of descriptors should be optional for patients to use as needed.

Providing an inner structure, enabling annotations, and describing collections can improve information access and expedite decision-making. Powerful search engines can use these metadata to allow patients direct and easy access not only to individual collections but also to their specific contents.

While these features can enhance collections’ actionability, it is important to note that collections are not meant for making independent clinical decisions by patients. Collections should be verified by a physician to serve as reliable tools for sensemaking and health self-management. However, collections can always be invaluable tools for patients to understand their health; organize thoughts, hypotheses, and insights; and communicate effectively with their physicians.

#### Collection Use for Patient-Provider Communication

As observed in our findings, patients can use collections to prepare for a clinical visit by devising checklists and organizing thoughts supported by evidence. During the visit, collections can be used for *note taking* and, afterward, for *recording reminders* and *follow-up actions*. These uses indicate how collections can start addressing known challenges during clinical visits, such as problem presentation [46], information retention [47], setting common ground, aligning goals, and understanding instructions [48]. To effectively tackle such challenges, the Collections feature should support richer note capturing and collaborative data analysis in a collocated setting [49,50].

Future improvements in capturing PGD could make collections more appealing to physicians. For physicians, PGD play a crucial role in understanding the boundaries and context for accurate diagnosis and optimal treatment [51]. However, physicians often face problems with PGD, such as incomplete data, inconsistent data structures, and insufficient time for reviewing due to poor organization [52]. These issues arise because patients use disjointed platforms to log their data, lacking consistent models for logging different types of data [52,53], and face challenges in efficiently using these platforms [42,52]. Collections can help overcome these issues by providing a single platform for logging PGD for various health issues in a simple, universal way that allows for flexibility, organization, and standardization.

An alternative approach to enhancing collections as a communication tool and fostering physician collaboration in their creation and verification is to introduce them as a *shared resource* similar to Google Docs [54,55]. While this may seem unconventional, it builds on the principles of OpenNotes [56]. OpenNotes provides access to and transparency regarding clinical notes, enabling patients to improve their treatment and EHR data quality by taking an active role in detecting errors, raising concerns, asking questions, or seeking clarifications [56,57].

Similar to OpenNotes, *shared collections* would follow the principle of asynchronous communication and transparency. However, shared collections could eliminate the expressiveness constraints and lack of efficient ways to provide granular and tailored context observed in existing messaging systems [58,59]. In addition, shared collections would enable direct editing of underlying data in collaborative ways, minimizing communication overhead.

Moreover, shared collections would introduce a new communication channel between patients and providers outside the traditional patient portal. Synchronizing the digital traces of care in collections with the provider’s EHR system to avoid discrepancies and legal issues should be a top consideration in future design iterations of the shared collection concept.

#### A Glimpse Into the Future: Collections and Generative Artificial Intelligence

In the future, we should explore the potential of generative artificial intelligence (GenAI) models to support patient sensemaking through collections. Tools such as ChatGPT [60] and Med-PaLM [61], which have demonstrated substantial medical knowledge [62-64], can replace the need for custom-made machine learning algorithms for knowledge-intensive tasks.

In particular, GenAI tools can aid in automatic and iterative collection construction with explanations and guidance. They can analyze the data within collections for insights, including medical records, PGD, notes, and tags. In addition, GenAI can assist in composing case narratives and talking points for clinical encounters. By offering this level of automation, GenAI can help tackle the significant knowledge challenges while lowering the labor barrier for patients’ sensemaking activities.

Using GenAI models, collection construction could rely on natural language instructions such as the following: “Group my EHR data by condition,” “Find all records related to my bronchitis,” or “Identify records that don’t belong to this collection and those that are missing.” GenAI models could also deliver context, explaining why certain records are included or excluded and providing educational material such as term definitions and clinical implications.

In addition, patients can issue commands for identifying relationships within their annotated data, such as “List all triggers for my headaches over the last year.” Finally, they can ask for help in constructing case presentations for clinical visits (eg, “Based on my ‘High Blood Pressure’ collection notes, write a 100-word summary”).

To be useful for sensemaking, GenAI tools do not need to achieve complete accuracy. While still striving for maximum reliability, their main value should come from providing an environment that enables and encourages patients to refine artificial intelligence–generated outputs. As such, the contribution of GenAI toward sensemaking would be evaluated on its ability to help the patient efficiently produce a satisfactory solution with minimal physician input.

Finally, existing approaches for supporting sensemaking through search and interactive visualizations should not be disregarded. Exploring the integration of GenAI, search, and visualization is a prudent strategy as different sensemaking tasks related to collection assembly, editing, and analysis may require diverse approaches based on complexity, patient skills, and artificial intelligence reliability.

### Limitations

This study has several limitations. First, the cohort skewed younger, likely due to recruitment via Craigslist (less popular with older adults) and the complexity of the remote setup. Second, participants used data from a fictitious patient, which may have reduced their motivation to learn the app and their ability to suggest real-life use cases. Third, participants had limited time to learn how to interact with collections, possibly affecting their perceptions of usability and utility. Future studies should have participants use their own data with automatic interaction logging. Despite these limitations, this study provided valuable insights into designing patient-facing sensemaking tools for organizing and augmenting EHR data.

### Conclusions

Collections can potentially improve patient-centered care by involving patients more in decision-making and encouraging self-advocacy. Current assumptions often expect patients to have the necessary skills, tools, and motivation. We believe that collections can lower these barriers, encouraging patients to *increase engagement* with their health data, better *educate themselves*, and *communicate more effectively* with their care providers.

Our study suggests that EHR data can be better used and more useful for patients through *improved organization* and *annotation*. This approach can incentivize patients to engage more deeply with their EHR data, develop insights, and reflect on their experiences. Patients felt that this empowered their awareness, resourcefulness, and proactivity regarding health issues, making them more prepared and better informed for clinician interactions.

These findings support our premise that collections are a crucial step toward *patient empowerment* and *self-advocacy*. With appropriate improvements, collections can enhance patients’ expertise by facilitating sensemaking activities and enabling insightful discussions with their physicians. First, collections motivate patients to construct health models based on their issues and ongoing problems. Second, patients gain medical education by actively participating in the evolution of collections through independent or system-assisted assembly and editing. Finally, patients acquire additional medical knowledge by engaging in meaningful discussions with their physicians and considering their feedback on collection verification.

Our study highlighted the importance of integrating PGD with EHR data. We envision a synergy in which patients use clinical data as a foundation, augmenting them with their observations, notes, and annotations to create personalized health narratives that support better health management and provider communication.

In the future, we should explore GenAI models to support patient sensemaking through collections. These models could help patients build collections, analyze the data within them, and produce health narratives more efficiently. Such enhancements may also reduce physicians’ workload for verifying collection contents, leading to more focused, evidence-driven discussions during visits.

Promising ideas from this work should be advanced carefully, with gradual design improvements tested in real-life settings. Respecting existing clinical practices and workflows can facilitate quicker adoption and more significant changes in the future. We believe that collections can revolutionize how patients interact with their medical records and communicate with their providers.

## Appendix

Multimedia Appendix 1 Demographic and participant characterization questions and the evaluation tasks for the 2 study sessions—session 1 and session 2.

## Abbreviations

EHR: electronic health record
FHIR: Fast Healthcare Interoperability Resources
GenAI: generative artificial intelligence
PGD: patient-generated data
RQ: research question
