# Supplementary material for: Patient-Generated Collections for Organizing Electronic Health Record Data to Elevate Personal Meaning, Improve Actionability, and Support Patient–Health Care Provider Communication: Think-Aloud Evaluation Study
Source: JMIR Hum Factors. 2025 Feb 3;12:e50331. doi: 10.2196/50331 (PMC11833264; doi:10.2196/50331)
Supplement: Multimedia Appendix 1 [file humanfactors_v12i1e50331_app1.docx]

**Study interview guide**

**SESSION 1**

Table S1. Demographic questions to paint the participant as a digital health consumer.

| **Demographics questions (5min)** |
| --- |
| 1. What is your age? 2. What is your highest level of education?    1. Does your job involve data analytics of some sort? 3. How would you describe your medical history – have you been seeing doctors a lot or not?    1. How many chronic conditions do you have? Anything that makes you monitor your health more closely and have more frequent doctor’s visits over a longer period of time qualifies.    2. How long have you had each of those conditions? 4. How many different providers have medical information about you? 5. How do you access the information from those providers?    1. How frequently do you use those Patient Portals or apps?    2. How hard would you say it is to keep track of your medical information from your providers?    3. What would be the biggest barrier for doing that? 6. Do you use Apple Health Records or any other third-party app to access your medical records from one or multiple providers?    1. Why yes, why no? 7. Do you currently use any devices or apps to keep track and make sense of your health/medical information?    1. What do you like and dislike about them? |

Table S2. Evaluation tasks for Session 1.

| **Session 1 (usability): familiarizing with and evaluating the interactions for Collection creation and building (50min)** |
| --- |
| Creating a Collection (5min) |
| 1. For the purposes of learning the basic mechanics around building a Collection from scratch, first Create a new Collection and name it “Toy Collection”. 2. Now, let’s add some descriptors about the Collection.    1. Please add a purpose to the Collection, something related to learning about this app.    2. Now, add a couple of tags to further describe, summarize or annotate the Collection for future quick access.    3. Finally, specify the priority of this Collection by marking it as urgent. 3. What was your experience with creating the new Collection?    1. How intuitive was it? Have you seen similar interactions elsewhere?    2. How useful do you find the descriptors for the Collection? 4. Now, let’s go back to the list of Collections. Let me know how can you see the details about the Collection you just created?    1. How intuitive was it? 5. What are possible improvements? |
| First impression (5min) |
| 1. Now, you will get familiarized with the user interface for exploring your data, finding patterns and saving relevant Records in the Collection. To do that, tap on the “Toy Collection”. 2. Please explain what do you see in the interface? 3. How overwhelming does it feel? |
| Data Model (5min) |
| What are the different Record categories you have?   1. What are the different types of Vital Signs? 2. How many Records for Blood Pressure do you have?    1. When is the latest one?    2. When is the earliest one? 3. How comfortable do you feel with this model? How close does this model come to how you think about your medical records? |
| Scoping (5min) |
| 1. Limit the Records you want to see by eliminating Care Plans, Diagnostic Reports and Encounters from the view. 2. Define a new time window, from January 1st 2010 to January 1st 2015, for which you want to see Records. 3. What was your experience with the scoping?    1. How intuitive was it? Why?    2. How familiar is the scoping mechanism to you? Have you seen similar scoping interactions elsewhere? 4. What are possible improvements? |
| Timeline (5min) |
| 1. Can you explain the Timeline visualization to me? 2. What do the bars represent?    1. In what period of time did you have most of your Records?    2. What does the horizontal line represent? 3. What do the triangles above the bars represent?    1. For how much of the time did you have a normal number of Records?    2. Was there any time that you had an extremely high number of Records? 4. What was your experience with the Timeline?    1. Was it understandable?    2. Does it give you the insights you need? 5. What are possible improvements? |
| Highlighting in the Visualization (5min) |
| 1. Highlight all your Conditions on the Timeline. 2. Highlight all your Immunizations on the Timeline. 3. Highlight any three Blood Pressure Records with systolic value above 125 on the Timeline.    1. Can you make the one with the highest value stand out? 4. Can you notice any patterns in the highlights? 5. What was your experience with highlighting the Records on the Timeline?    1. How intuitive was it? Why?    2. How familiar is the highlighting mechanism to you? Have you seen similar highlighting interactions elsewhere? 6. What are possible improvements? |
| Periodicity, cooccurrence, pre-post (5min) |
| 1. How frequently do you have a flu shot? 2. How does this frequency compare to Zoster? 3. Please clean up the highlights you made. On how many occasions did you have high blood pressure – 125 or more, and high BMI – 30 or more, around the same time? 4. Please clean up the highlights you made. Did you have any respiratory conditions after you took Amoxicillin? |
| Saving in Collection (5min) |
| 1. We will just randomly save Records to the Collection. There is no meaning to why they are grouped together. The main purpose is to see how you can execute the saving task.    1. Save all Body Weight Records in the Collection.    2. Save any three Blood Pressure Records in the Collection.    3. Save any two Conditions and any two Lab Results in the Collection. 2. What was your experience with saving the Records in a Collection?    1. How intuitive was it? Why?    2. How familiar is the saving mechanism to you? Have you seen similar saving interactions elsewhere? 3. What are possible improvements? |
| Reviewing a Collection (5min) |
| 1. Swipe left, to go to the view that allows you to review the Collection. 2. Explain what you see in the interface? 3. Do you find these sortings useful?    1. For what purposes would you use each of the sortings?    2. Is there some other type of sorting that needs to be here? Why? 4. What else would you like to be able to do in this view? |
| Feedback (5min) |
| 1. What was your impression of this app?    1. What did you like?    2. What did you dislike? 2. How intuitive was the app? 3. How easy or hard was it to explore the data? 4. How useful were the features in the app to identify patterns in the data? 5. How did you like the mechanism for saving Records in the Collection? 6. What are some improvements you would like to see? |

**SESSION 2**

Table S3. Evaluation tasks for Session 2.

| **Session 2 (usefulness): building and annotating a more realistic Collection and possible use-cases (45min)** |
| --- |
| Building a Collection (15min) |
| 1. You will now create a Collection that is more realistic and meaningful for use in a real-life scenario. We will assume that you are preparing for an upcoming visit to your physician’s office related to potential issues with high blood pressure. Create a collection called “High Blood Pressure”.    1. Add the purpose for the Collection    2. Add a few tags    3. Mark its priority 2. Add the Records with blood pressure with systolic value over 120, and body mass index over 30. 3. What was your experience with assembling the Records for the Collection?    1. How laborious was it?    2. What are some ways in which we can make this assembling process more efficient?    3. How do you feel about having the system prepopulate the Collection for you, and let you modify it afterwards? |
| Finding insights from a Collection (5min) |
| 1. Let’s see if you can notice any interesting patterns in the Records you just assembled. For that purpose, please go to the Review screen for your Collection. Can you notice any patterns, some relationship between the high blood pressure and high body mass index? |
| Annotating Collections and Records (10min) |
| 1. For the pattern you just observed, please add a note to the Collection, something you can use in your upcoming clinical visit. 2. What was your experience with adding the notes?    1. How intuitive was it? Why?    2. How familiar was this notes adding mechanism to you? Have you seen similar interactions elsewhere? 3. You can also add notes to individual Records. Find the Record with the highest blood pressure and add a note to it, something that is important for context, but was not captured by the EHR system. 4. What was your experience with adding the notes?    1. How intuitive was it? Why?    2. How familiar was this notes adding mechanism to you? Have you seen similar interactions elsewhere? 5. Do you need additional types of annotations for the Collection or the individual Records to add more context or meaning to it?    1. What are those? |
| Searching the Collections (5min) |
| As the number of Collections grows, accessing them quickly becomes more important. We provide a search feature that lets you search by title, purpose, notes, tags, and priority.   1. What is your opinion on these searching capabilities? 2. How else would you like to be able to filter or search your Collections? |
| Feedback (10min) |
| 1. How useful do you think the Collections can be for you? 2. What are some use-cases for the Collections that you can think of? 3. What are some improvements you would like to see for the Collections?    1. Automatic support for building Collections?    2. Automatically finding data patterns in the Collections?    3. Patient generated data? |

## 
